# CTRP3 ameliorates cerulein-induced severe acute pancreatitis in mice via SIRT1/NF-κB/p53 axis

**DOI:** 10.1042/BSR20200092

**Published:** 2020-10-13

**Authors:** Chunyang Lv, Yuan He, Mingli Wei, Guiyun Xu, Chuang Chen, Zhen Xu, Zhilong Ding

**Affiliations:** 1 Department of Hepatobiliary Surgery, The Affiliated Huai’an Hospital of Xuzhou Medical University and The Second People’s Hospital of Huai’an, Huai’an 223002, China; 2 Department of General Surgery, Heping Hospital, Changzhi Medical College, Changzhi 046600, China

**Keywords:** CTRP3, NF-κB signaling, p53 acetylation, severe acute pancreatitis, SIRT1

## Abstract

Severe acute pancreatitis (SAP) is a common and life-threatening clinical acute abdominal disease. C1q/tumor necrosis factor-related protein 3 (CTRP3), a novel paralog of adiponectin, has been identified as a crucial regulator in multiple types of inflammatory disorders. However, the biological role of CTRP3 in SAP remains poorly understood. The present study aimed to characterize the role of CTRP3 in SAP and illuminate the potential mechanisms involved. In the current study, SAP mouse models were induced by seven hourly intraperitoneal injection of cerulein (50 μg/kg) and an immediate intraperitoneal injection of lipopolysaccharide (10 mg/kg) after the last cerulein administration. Histological examination and serological analysis demonstrated that SAP mouse models were successfully established. Herein, we found that CTRP3 expression was significantly decreased in the pancreatic tissues of SAP mice compared with normal control mice. Furthermore, we explored the effects of CTRP3 rescue in SAP mice and discovered that CTRP3 overexpression attenuated pathological lesions, inhibited inflammatory mediator release and repressed acinar cell apoptosis. Notably, mechanistic studies revealed that CTRP3 overexpression suppressed NF-κB p65 phosphorylation and p53 acetylation to alleviate cerulein-induced SAP in mouse models through activation of silent information regulator 1 (SIRT1), a nicotinamide adenine dinucleotide-dependent protein deacetylase. Collectively, our data indicate that CTRP3 may exert its protective effects in SAP mice via regulation of SIRT1-mediated NF-κB and p53 signaling pathways, implying a promising therapeutic strategy against SAP.

## Introduction

Acute pancreatitis (AP) is a common acute clinical abdominal disorder that is characterized by acinar cell necrosis and abnormal inflammatory response [1–3]. It is estimated that approximately 250000 patients are diagnosed with AP in the United States annually [4,5]. Besides, the incidence of AP in the U.K. is reported to be approximately 30 per 100000 per annum, which is linked to gallstones and excessive alcohol consumption to some extent [6–8]. Severe AP (SAP), the most serious pathological type of AP, accounts for roughly 15% of all AP cases [9,10]. It is well documented that SAP is characterized by a rapid onset and a high mortality [11,12]. It is widely acknowledged that activation of NF-κB signaling pathway could induce inflammatory response during the pathogenesis of AP [13–15]. Furthermore, it is reported that p53 acetylation could be capable of suppressing cell proliferation and survival, which would contribute to development of AP [16]. Despite the fact that great progress has been achieved regarding the diagnosis and management of SAP, its mortality rate has not declined significantly over the past several decades. There is no doubt that SAP remains a huge challenge around the world. Therefore, it is of critical importance to unveil the potential molecular mechanisms underlying SAP development and develop effective therapeutic strategies for SAP patients.

C1q/tumor necrosis factor-related protein 3 (CTRP3), a novel adipokine, belongs to the highly conserved CTRP superfamily of adiponectin paralogs [17,18]. Previous studies have demonstrated that CTRP3 is an endogenous antagonist of lipopolysaccharide [19] and that aberrant CTRP3 expression is related to multiple types of human disorders, such as arthritis [20], sepsis [21] and myocardiac dysfunction [22]. Nonetheless, the biological role of CTRP3 in SAP and potential molecular mechanism remain largely unclear. It is widely acknowledged that acinar cell damage, excessive inflammatory response and oxidative stress are crucial events during SAP development [23,24]. Silent information regulator 1 (SIRT1), a nicotinamide adenine dinucleotide-dependent protein deacetylase, has been reported to exert protective roles against inflammatory diseases via regulation of some key signaling pathways [25–27]. In addition, a recent study has revealed that CTRP3 could protect against cardiac injury through activation of SIRT1 [28].

The purpose of the present study was to explore the role of CTRP3 in cerulein-induced SAP mice and clarify the potential molecular mechanisms involved. Based on the above evidence, we established SAP models in mice and characterized the expression pattern of CTRP3 in the pancreas of SAP mice. Moreover, functional studies and mechanistic studies were subsequently carried out. Collectively, the current study may offer some insights for the protective effects of CTRP3 against cerulein-induced SAP in mice.

## Materials and methods

### Animals

Male ICR mice with body weight of 25–30 g were purchased from Shanghai Laboratory Animal Co. Ltd. (Shanghai, China). All the animals were housed in a specific pathogen-free (SPF) environment ICR mice were kept at 20–25°C with a relative humidity of 50–55% and a 12/12-h light/dark cycle. The animal experiments were carried out in the Affiliated Huai’an Hospital of Xuzhou Medical University (Huai’an, China). All the experimental procedures were approved by experimental Animal Ethics Committee of the Affiliated Huai’an Hospital of Xuzhou Medical University.

### Establishment of SAP animal models

To construct SAP animal models, mice (*n*=5) were given seven hourly intraperitoneal injections of cerulein (50 μg/kg), followed by an immediate intraperitoneal injection of LPS (10 mg/kg) after the last cerulein administration. Normal control mice (*n*=5) were treated with the same volumes of saline. All the animals were anesthetized using isoflurane and killed via carbon dioxide inhalation at 8 h after the final injection. Then blood samples and the fresh pancreas were collected for characterization of SAP models.

### Adeno-associated viral vector construction

The recombinant adeno-associated viral vector carrying CTRP3 (AAV-CTRP3) or green fluorescent protein (negative control adeno-associated viral vector (AAV-NC)) was constructed by Shanghai Genepharma Co. Ltd. (Shanghai, China). The adeno-associated viral vector carrying green fluorescent protein (AAV-NC) was used as the control.

### Experimental grouping design

A total of 20 ICR mice were randomly allocated into four experimental groups (*n*=5): (1) normal control group receiving saline; (2) SAP group; (3) SAP+AAV-NC group; (4) SAP+AAV-CTRP3 group. For SAP+AAV-NC group and AAV+CTRP3 group, mice were injected with 200 μl of AAV-NC and AAV-CTRP3 (2 × 10^9^ PFU/ml) via tail vein at 6 h post-SAP model establishment. After 72 h, all the mice were anesthetized using isoflurane and killed via carbon dioxide inhalation. And blood and pancreas samples were immediately collected for further analysis.

### Histological examination

Fresh pancreatic samples were fixed in 4% paraformaldehyde, embedded in paraffin and cut into 5-μm slices. Paraffin sections of pancreatic tissues were then subjected to Hematoxylin and Eosin (H&E) staining for histological examination in accordance with the standard procedures. Images were captured under a light microscope. All the assessments were carried out by two experienced pathologists blindly.

### Real-time quantitative PCR analysis

Total RNA was extracted from pancreatic tissues using the TRIzol reagent (Takara Bio, Dalian, China) and reverse-transcribed into complementary DNA (cDNA) using the PrimeScript™ RT reagent kit (Takara) in line with the manufacturer’s instructions. Real-time PCR was carried out using SYBR Premix Ex Taq™ Kit (Takara) on an ABI 7500 real-time PCR system (Life Technology, Carlsbad, CA, USA). All the samples were analyzed in triplicate. The relative mRNA expression level was calculated using the 2^−ΔΔ*C*_t_^ method and normalized to β-actin. The specific primers were synthesized by Shanghai Sangon Biotech Co. Ltd. (Shanghai, China). The sequence information of the specific primers were listed as follows: CTRP3, forward 5′-ATGGAGGTGAGCAGAAGAGC-3′ and reverse 5′-CACAGTCCCCGTTTTAGCAT-3′; β-actin, forward 5′-GATCATTGCTCCTCCTGAG-3′ and reverse 5′-ACTCCTGCTTGCTGATCCAC-3′.

### Cell transfection

Cell transfection was performed using Lipofectamine 2000 (Invitrogen, Carlsbad, CA, U.S.A.) based on the instructions of the manufacturer. Short hairpin RNA specifically targeting CTRP3 (shCTRP3) was designed and synthesized by Shanghai GenePharma Co. Ltd. (Shanghai, China). The sequence information of shCTRP3 was listed as follows: 5′-CACCGCAACACAGTCTTCAGCATGTCGAAACATGCTGCTGAAGACTGTGTTGC-3′. EX527 (a specific SIRT1 inhibitor) and SRT1720 (a specific SIRT1 activator) were purchased from SelleckChem Corp (Houston, TX, U.S.A.).

### Western blotting analysis

The pancreas samples were subjected to 10% SDS/PAGE and transferred on to the PVDF membrane (Bio-Rad, Hercules, CA, U.S.A.). Subsequently, the PVDF membranes were blocked using 5% fat-free milk at room temperature for 1 h at room temperature and incubated with the following primary antibodies: anti-β-actin (1:3000, ab8227), anti-CTRP3 (1:3000, ab36870), anti-SIRT1 (1:3000, ab110304), anti-p-NF-κB p65 (1:3000, ab86299), anti-NF-κB p65 (1:3000, ab16502) and anti-acetyl-p53 (1:3000, ab75754) at 4°C overnight. After washing with TBST three times, slides were incubated with horseradish peroxidase–conjugated secondary antibodies at room temperature for 1 h. The signals were developed using ECL Plus detection System (Amersham, Piscataway, NJ, U.S.A.) and quantified using ImageJ software (NIH, Bethesda, MD, U.S.A.).

### Serum amylase and lipase activity analysis

To measure serum amylase and lipase activities, sera were prepared from blood samples using centrifugation. Serum amylase and lipase activities were measured using amylase assay kit and lipase assay kit (Nanjing Bioengineering Corp, Nanjing, China), respectively. All the procedures were performed according to the manufacturer’s instructions.

### Enzyme-linked immunosorbent assay

To evaluate inflammatory cytokines (tumor necrosis factor α (TNFα), interleukin (IL) 1β (IL-1β), IL-6 and IL-10) in the serum and pancreas samples, commercial Enzyme-linked immunosorbent assay (ELISA) kits (Millipore, Billerica, MA, U.S.A.) were applied based on the manufacturer’s protocols.

### Measurement of nitric oxide concentration and nitric oxide synthase activity

To assess nitric oxide (NO) concentration and NO synthase (NOS) activity in the serum and pancreas samples, NO assay kit and NOS assay kit (Nanjing Bioengineering Corp, Nanjing, China) were used, respectively. All the procedures were done according to the manufacturer’s instructions.

### Measurement of myeloperoxidase and superoxide dismutase activities

To evaluate pancreatic myeloperoxidase (MPO) and superoxide dismutase (SOD) activities, pancreas tissue samples were thawed and homogenized in normal saline. MPO and SOD activities in the tissue homogenate were measured using MPO activity kit and SOD activity kit in accordance with the manufacturer’s instructions (Nanjing Jiancheng Bioengineering Corp., Nanjing, China), respectively.

### Immunohistochemical staining

Paraffin slides of pancreatic tissues were de-paraffinized and rehydrated using xylene and graded alcohols. After antigen retrieval using critic acid, endogenous peroxidase was blocked using 3% H_2_O_2_. Afterward, the slides were incubated with specific primary antibodies at 4°C overnight. Anti-MPO (ab139748) and anti-p-NF-κB p65 (ab86299) were purchased from Abcam Company. After washing three times with PBS, the sections were incubated with secondary antibody for 1 h at 37°C. The slides were stained using diaminobenzidine (DAB). Integral optic density (IOC) of images was evaluated using Image-Pro Plus 6.0 software (Media Cybernetics, Rockville, MD, U.S.A.).

### Statistical analysis

Statistical analysis was performed using SPSS 20.0 software (SPSS Inc., Chicago, IL, U.S.A.). Results were expressed as mean ± standard deviation. The difference between two groups was assessed by Student’s *t* test. The difference among three or more groups was compared using one-way ANOVA. A *P*-value of <0.05 was considered statistically significant.

## Results

### CTRP3 down-regulation is observed in the pancreas of cerulein-induced SAP mice

**Figure 1:**
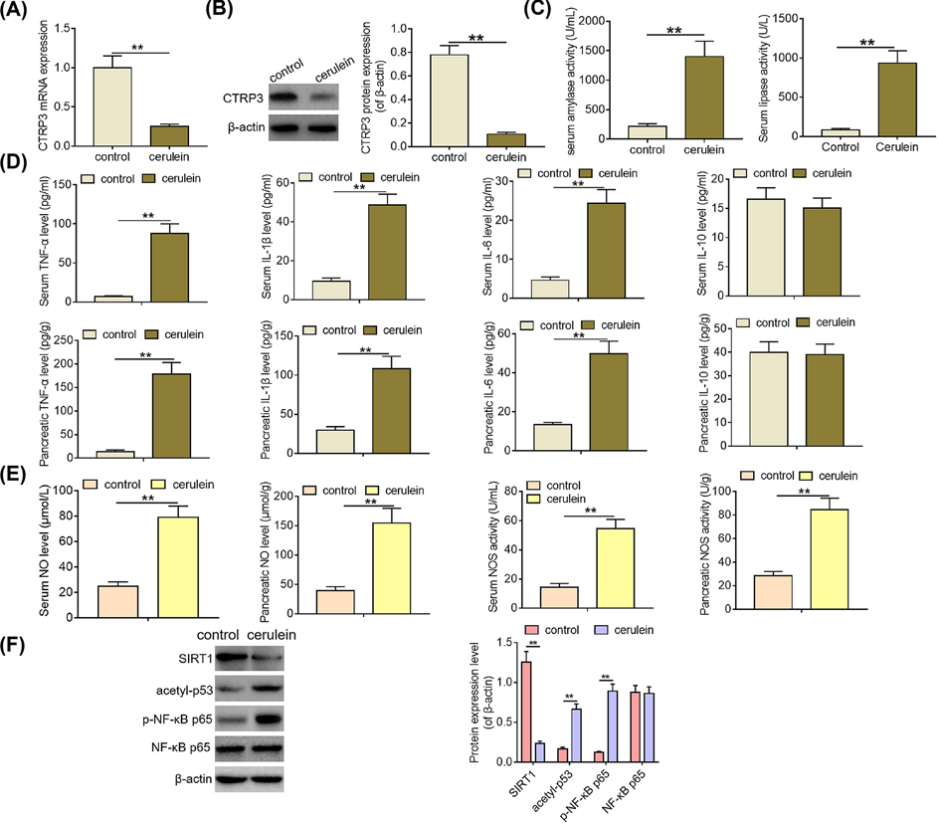
CTRP3 down-regulation is observed in the pancreas of cerulein-induced SAP mice (**A**) CTRP3 mRNA expression levels were determined by qPCR analysis in the pancreas of mice from normal control group (*n*=5) and SAP group (*n*=5). (**B**) CTRP3 protein expression levels were examined by Western blotting analysis in the pancreas of mice from normal control group and SAP group. (**C**) Serum amylase and lipase activities of mice from normal control group and SAP group were measured using amylase assay kit and lipase assay kit, respectively. (**D**) The levels of inflammatory cytokines (TNFα, IL-1β, IL-6 and IL-10) were determined using ELISA in the serum and pancreas of mice from normal control group and SAP group. (**E**) NO concentration and NOS activity in the pancreas and serum of mice from normal control group and SAP group were detected via NO assay kit and NOS assay kit, respectively. (**F**) The protein expression levels of SIRT1, acetylated p53 and phosphorylated NF-κB p65 were examined by Western blotting analysis in the pancreas of mice from normal control group and SAP group. **P<0.01.

In the present study, SAP models in mice were established by seven hourly intraperitoneal injection of cerulein (50 μg/kg) and an immediate intraperitoneal injection of lipopolysaccharide (10 mg/kg) after the last cerulein administration in mouse. First, we examined the expression patterns of CTRP3 in the pancreatic tissues via qRT-PCR analysis and Western blotting analysis. As shown in Figure 1A,B, CTRP3 expression was significantly down-regulated in the pancreatic tissues of SAP mice compared with normal control mice. Subsequently, we measured serum amylase and lipase activities, two crucial indicators used to evaluate SAP, in the control normal and SAP mice. A remarkable increase in serum amylase and lipase activities was observed in SAP mice compared with normal control mice (Figure 1C). Moreover, the levels of inflammation-related cytokines (TNFα, IL-1β, IL-6 and IL-10) and NO as well as the activity of NOS in the serum and pancreatic tissues were also analyzed using ELISA, NO assay kits and NOS assay kits, respectively. A notable increase in TNFα, IL-1β, IL-6 and NO levels was found in the serum and pancreatic tissues of SAP mice in comparison with normal control mice (Figure 1D,E). Furthermore, the serum and pancreatic tissues of SAP mice also exhibited higher NOS activity than normal control mice (Figure 1E). No significant differences about serum and pancreatic IL-10 concentrations were observed between two groups (Figure 1D). Previous studies have reported that CTRP3 could alleviate cell death and inflammation via activating SIRT1 and that SIRT1 could modulate NF-κB and p53 signaling. As shown in Figure 1F, SIRT1 expression was notably increased in the pancreatic tissues of SAP mice in comparison with normal control mice. Furthermore, Western blotting analysis demonstrated a significant increase in the phosphorylation of NF-κB p65 and acetylation of p53 in the pancreas of SAP mice compared with normal control mice. Taken together, these results indicate that CTRP3 may play a crucial role in the development of SAP.

### CTRP3 overexpression alleviates pancreatic injury in cerulein-induced SAP mice

**Figure 2:**
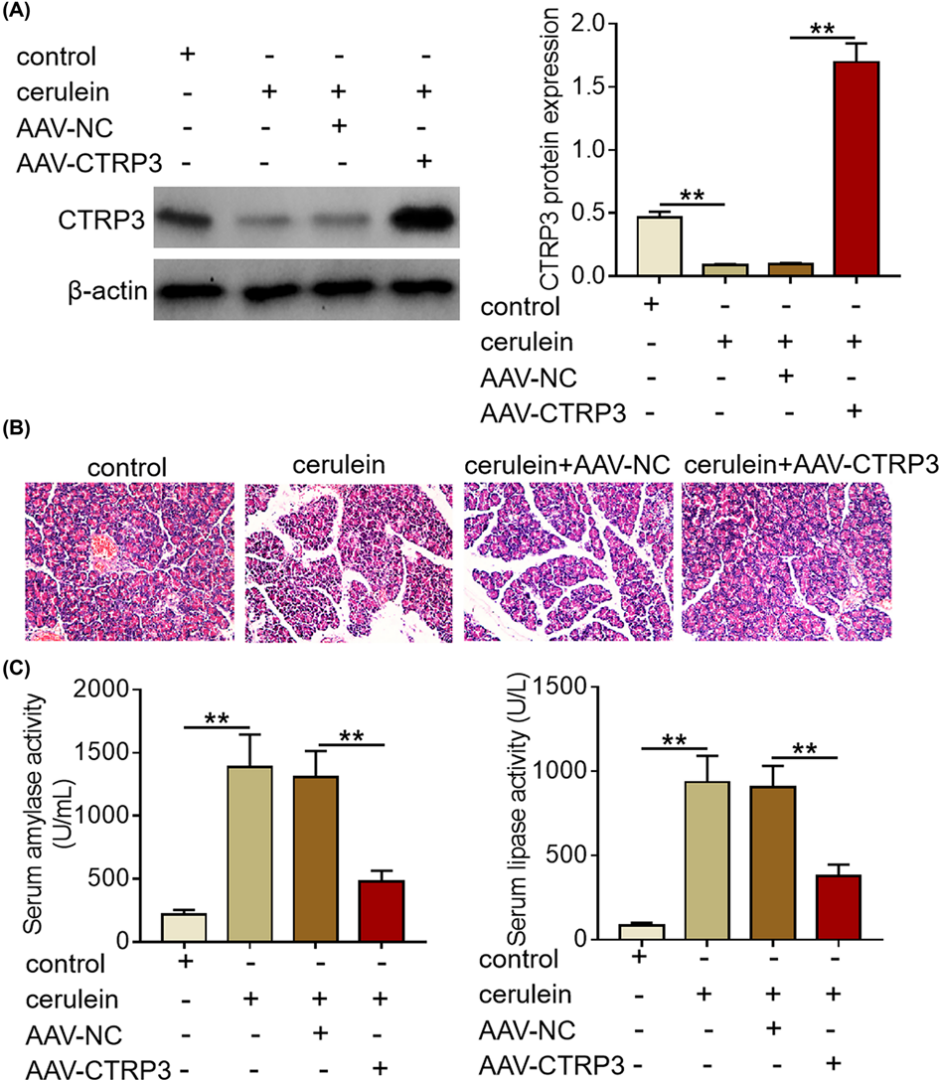
CTRP3 overexpression alleviates pancreatic injury in cerulein-induced SAP mice (**A**) CTRP3 protein expression was examined by Western blotting analysis in the pancreas of mice from different treatment groups after tail intravenous injection of AAV-NC or AAV-CTRP3. (**B**) Representative images of H&E staining of pancreatic tissues of mice from different treatment groups (magnification, 100×). (**C**) Serum amylase and lipase activities of mice from different treatment groups were determined by amylase assay kit and lipase assay kit, respectively. **P<0.01.

To explore the biological role of CTRP3 in SAP, we rescued the expression of CTRP3 in mice via tail intravenous injection of adeno-associated virus vectors carrying *CTRP3* gene (AAV-CTRP3). Western blotting analysis revealed that transfection of AAV-CTRP3 restored CTRP3 expression in the pancreas of SAP mice (Figure 2A). Furthermore, the results of the histological examination showed that remarked acinar cell degeneration and necrosis were observed in the pancreas of SAP mice, while no significant pathological changes were found in the pancreas of mice from control group and CTRP3 overexpression group (Figure 2B). In addition, we evaluated serum amylase and lipase activity of mice from different groups. Moreover, CTRP3 overexpression abrogated the increase in serum amylase and lipase activity induced by cerulein exposure (Figure 2C). To sum up, these findings provides some clues that CTRP3 overexpression could ameliorate pancreatic injury in cerulein-induced SAP mice.

### CTRP3 overexpression reduces the production of pro-inflammatory mediators in SAP mice

**Figure 3:**
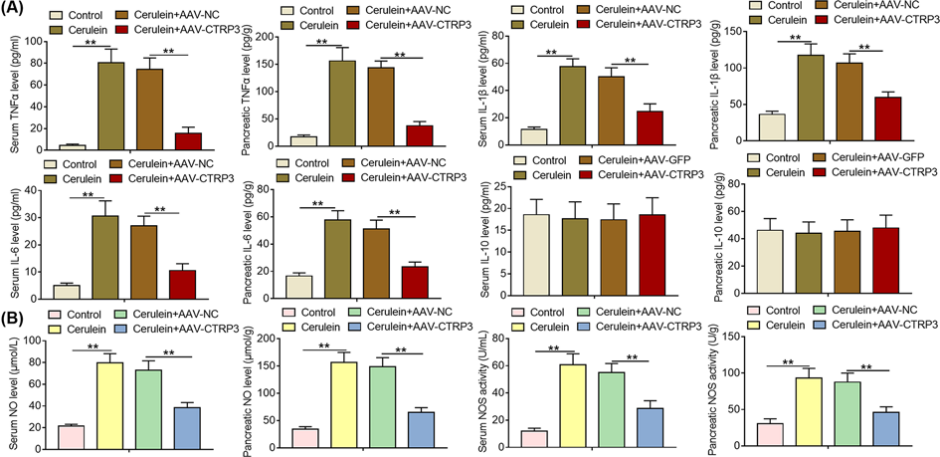
CTRP3 overexpression reduces the production of pro-inflammatory mediators in SAP mice (**A**) The levels of inflammatory cytokines (TNFα, IL-1β, IL-6 and IL-10) were determined using ELISA in the serum and pancreas of mice from different treatment groups after tail intravenous injection of AAV-NC or AAV-CTRP3. (**B**) NO concentration and NOS activity in the serum and pancreas of mice from different treatment groups were detected via NO assay kit and NOS assay kit, respectively, after tail intravenous injection of AAV-NC or AAV-CTRP3. **P<0.01.

It is widely acknowledged that inflammatory mediators are crucial evaluation indicators for tissue damage and inflammatory disorders. Here, we assessed the effects of CTRP3 overexpression on the levels of some critical inflammatory mediators in the serum and pancreatic tissues of SAP mice. As evident from ELISA results, CTRP3 overexpression abolished the increase in TNFα, IL-1β and IL-6 levels of serum and pancreatic tissues induced by cerulein exposure (Figure 3A). Moreover, it was observed that CTRP3 overexpression reversed the increase in NO content and NOS activity of serum and pancreatic tissues of SAP mice (Figure 3B). To sum up, these results suggest that CTRP3 overexpression reduces the production of pro-inflammatory mediators in SAP mice.

### CTRP3 overexpression decreases MPO activity and increases SOD activity in SAP mice

**Figure 4:**
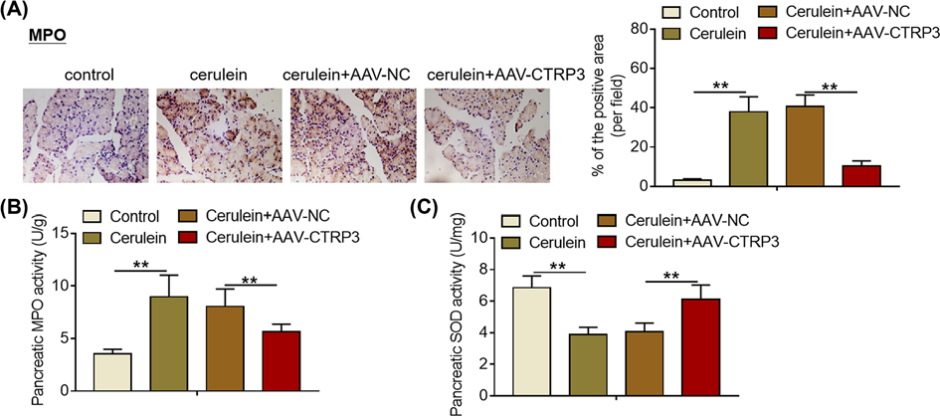
CTRP3 overexpression decreases MPO activity and increases SOD activity in SAP mice (**A**) MPO protein expression in the pancreas of mice from different treatment groups was visualized by immunohistochemical staining after tail intravenous injection of AAV-NC or AAV-CTRP3. (**B**) MPO activity in the pancreas of mice from different treatment groups was analyzed by MPO assay kit after tail intravenous injection of AAV-NC or AAV-CTRP3. (**C**) SOD activity in the pancreas of mice from different treatment groups was evaluated by SOD assay kit after tail intravenous injection of AAV-NC or AAV-CTRP3. **P<0.01.

It is well documented that MPO activity is a key indicator reflecting the accumulation of polymorphonuclear neutrophils in the pancreatic tissues [29,30]. Decreased SOD activity is considered to be closely related to oxidative stress [31,32]. Notably, elevated MPO activity and reduced SOD activity in the pancreas are important features during SAP development [33,34]. The results of the immunohistochemical staining revealed that more MPO-positive cells were observed in the pancreas of SAP mice compared with normal control group (Figure 4A). CTRP3 overexpression abrogated the increase in MPO-positive cells in the pancreatic tissues of SAP mice (Figure 4A). Furthermore, it was found that CTRP3 overexpression abolished the increase in pancreatic MPO activity induced by cerulein exposure (Figure 4B). Additionally, a dramatic decrease in pancreatic SOD activity was detected in SAP mice compared with normal control groups (Figure 4C). Notably, CTRP3 overexpression significantly attenuated the decrease in SOD activity of pancreatic tissues of SAP mice (Figure 4C). In sum, these findings indicate that CTRP3 overexpression decreases MPO activity and increases SOD activity in SAP mice.

### CTRP3 regulates p53 and NF-κB signaling pathways via SIRT1 in primary pancreatic acinar cells of mice

**Figure 5:**
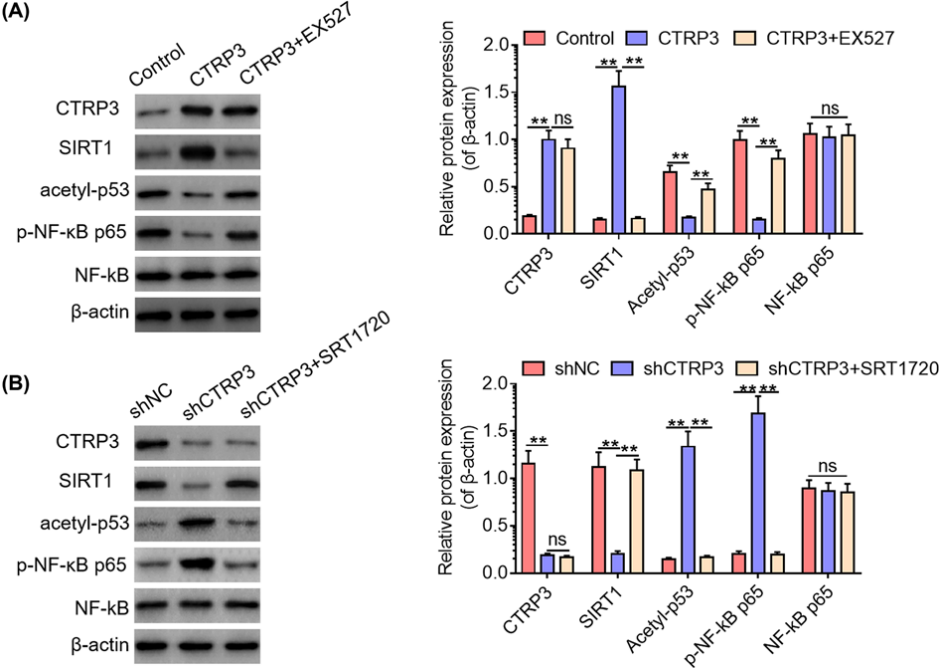
CTRP3 regulates p53 and NF-κB signaling pathways via SIRT1 in primary pancreatic acinar cells of mice (**A**) The protein expression levels of CTRP3, SIRT1, acetylated p53 and phosphorylated NF-κB p65 were examined by Western blotting analysis after transfection of SIRT1 specific inhibitor EX527 in primary pancreatic acinar cells treated with CTRP3 expression vectors. (**B**) The protein expression levels of CTRP3, SIRT1, acetylated p53 and phosphorylated NF-κB p65 were examined by Western blotting analysis after transfection of SIRT1-specific activator SRT1720 in shCTRP3-treated primary pancreatic acinar cells. **P<0.01; ns, not significant.

To clarify the possible molecular mechanisms underlying the alleviating effects of CTRP3 overexpression in cerulein-induced SAP, we carried out subsequent studies in primary pancreatic acinar cells of mice. First, we co-transfected primary pancreatic acinar cells with CTRP3 expression vector and EX527, a specific SIRT1 inhibitor [35,36], followed by Western blotting analysis. As shown in Figure 5A, CTRP3 overexpression significantly suppressed the phosphorylation of NF-κB p65 and the acetylation of p53, whereas EX527 reversed the inhibitory effects of CTRP3 overexpression on NF-κB and p53 signaling in primary pancreatic acinar cells. Furthermore, we also co-transfected primary pancreatic acinar cells with shCTRP3 and SRT1720, a specific SIRT1 activator [37,38]. Western blotting analysis showed that CTRP3 knockdown remarkably facilitated the phosphorylation of NF-κB p65 and the acetylation of p53, while SIRT1 activation abolished the promoting effects of CTRP3 ablation on NF-κB p65 phosphorylation and p53 acetylation in primary pancreatic acinar cells (Figure 5B). To sum up, the above findings manifest that CTRP3 regulates NF-κB and p53 signaling through activating SIRT1 in primary pancreatic acinar cells.

### CTRP3 modulates p53 and NF-κB signaling pathways via SIRT1 *in vivo*

**Figure 6:**
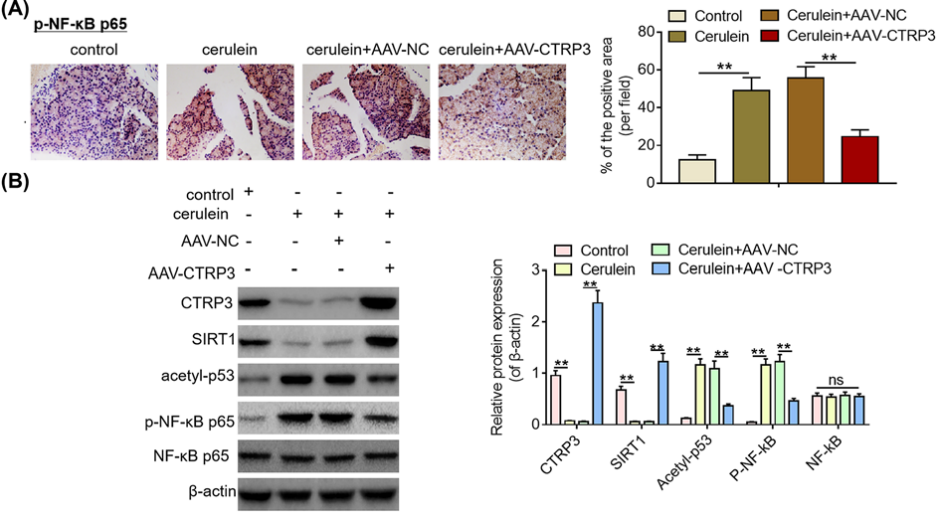
CTRP3 modulates p53 and NF-κB signaling pathways via SIRT1 *in vivo* (**A**) Immunohistochemical staining was used to visualize the protein expression of phosphorylated NF-κB p65 in the pancreas of mice from different treatment groups after tail intravenous injection of AAV-NC or AAV-CTRP3. (**B**) The protein expression levels of CTRP3, SIRT1, acetylated p53 and phosphorylated NF-κB p65 in the pancreas of mice from different treatment gruops were examined by Western blotting analysis after tail intravenous injection of AAV-NC or AAV-CTRP3. **P<0.01.

To further validate the putative molecular mechanisms *in vitro*, *in vivo analysis* was performed in cerulein-induced SAP mice. As displayed in Figure 6A, cerulein exposure remarkably increased p-NF-κB p65-positive cells in the pancreas of mice, whereas CTRP3 rescue could reverse the increase in p-NF-κB p65-positive cells. Subsequently, Western blotting analysis was used to evaluate the effects of CTRP3 rescue on SIRT1 expression, NF-κB p65 phosphorylation and p53 acetylation in the pancreas of cerulein-induced SAP mice. It was found that CTRP3 overexpression abrogated the inhibitory effect of cerulein exposure on SIRT1 protein expression in the pancreatic tissues of SAP mice (Figure 6B). Besides, CTRP3 rescue also reversed the promoting effects of cerulein exposure on NF-κB p65 phosphorylation and p53 acetylation in the pancreas of SAP mice, which was consistent with the observations *in vitro*. Taken together, these results indicate that CTRP3 could modulate SIRT1-mediated NF-κB and p53 signaling pathways *in vivo*.

## Discussion

SAP, a life-threatening clinical acute abdomen, has imposed enormous pressures on public health due to its high morbidity and mortality [39,40]. Nowadays, there is still a lack of effective therapeutic approaches for SAP. Hence, it is of vital importance to clarify the underlying molecular mechanisms and develop novel treatment strategies with high efficacy and minimal side effects. It is widely accepted that gene therapies may be promising approaches for SAP treatment [41,42]. Previous studies have demonstrated the protective effects of CTRP3 against multiple type of human disorders [20–22]; nonetheless, the biological role of CTRP3 in SAP and the possible mechanisms remain largely unclear.

In the current study, SAP mouse models were successfully constructed via cerulein injection. Furthermore, we characterized the expression patterns of CTRP3 in the pancreatic tissues and found that CTRP3 expression was significantly decreased in the pancreas of cerulein-induced SAP mice compared with normal control mice. In order to gain a better understanding of CTRP3, we rescued its expression in SAP mice through tail intravenous injection of AAV-CTRP3. Moreover, it was observed that CTRP3 overexpression could effectively ameliorate pancreatic damage and attenuate inflammatory status in cerulein-induced SAP mice, suggesting the protective effects of CTRP3 against SAP. To elucidate the potential protective mechanisms of CTRP3 against SAP, we carried out in-depth studies. In the previous reports, it was revealed that CTRP3 could alleviate organ injuries by activating SIRT1 [28]. In addition, it is well documented that SIRT1 may exert its functions through modulating the downstream signaling pathways [43–45].

In the present study, it was also found that SIRT1 expression was remarkably down-regulated in the pancreatic tissues of cerulein-induced SAP mice in comparison with normal control mice, which was in line with CTRP3 expression pattern. Moreover, our data demonstrated that rescue of CTRP3 could ameliorate cerulein exposure-induced SAP in mouse models via activation of SIRT1, which was consistent with previous findings about protective effects of CTRP3 [20–22]. In order to better illuminate the molecular mechanisms underlying the protective effects of CTRP3/SIRT1 axis, we evaluated two crucial signaling pathways, NF-κB pathway and p53 pathway. In the present study, a significant increase in phosphorylated NF-κB p65 level and acetylated p53 level was observed in the pancreas of cerulein-induced SAP mice compared with normal control mice.

Furthermore, subsequent mechanistic investigations implied that SIRT1 mediated the regulatory effects of CTRP3 on NF-κB p65 phosphorylation and p53 acetylation *in vitro*. Herein, it was found that specific inhibition of SIRT1 could reverse the suppressing effects of CTRP3 overexpression on NF-κB p65 phosphorylation and p53 acetylation and that specific activation of SIRT1 abolished the promoting effects of CTRP3 knockdown on NF-κB p65 phosphorylation and p53 acetylation *in vitro*. In addition, *in vivo* studies also demonstrated that rescue of CTRP3 alleviated pancreatic damage in SAP mice via modulating SIRT1-mediated NF-κB pathway and p53 pathway. It is widely acknowledged that NF-κB pathway activation is closely associated with inflammatory processes [46,47]. Of note, p53 acetylation is proposed to contribute to occurrence of tissue and organ injuries [48,49]. Taken together, these data indicated that CTRP3 exert its protective effects against cerulein-induced SAP in mice via SIRT1/NF-κB/p53 axis.

In summary, the present study for the first time revealed the protective effects of CTRP3 against cerulein-induced SAP in mice. Furthermore, current findings demonstrated that CTRP3 may exert its protective effects via regulation of SIRT1-mediated NF-κB and p53 signaling pathways. Collectively, our data provide novel insights into understanding the molecular mechanisms underlying SAP development, suggesting that CTRP3/SIRT1/NF-κB/p53 axis may represent a promising therapeutic target for SAP patients.

## Abbreviations

AAV-CTRP3: adeno-associated viral vector carrying CTRP3
AAV-NC: negative control adeno-associated viral vector
CTRP3: C1q/tumor necrosis factor-related protein 3
ELISA: enzyme-linked immunosorbent assay
IL: interleukin
MPO: myeloperoxidase
NO: nitric oxide
NOS: NO synthase
SAP: severe acute pancreatitis
shCTRP3: short hairpin RNA specifically targeting CTRP3
SIRT1: silent information regulator 1
SOD: superoxide dismutase
TNFα: tumor necrosis factor α
